# High‐Performance Oxidation and Nanomolar Detection of Phenylhydrazine Using a 6‐Hydroxyflavone‐Based Molecular Electrocatalyst Functionalized Multiwalled Carbon Nanotube in Batch Injection Analysis

**DOI:** 10.1002/open.202500140

**Published:** 2025-06-09

**Authors:** Valisireddy Lavanya, Kannappan Santhakumar, Annamalai Senthil Kumar

**Affiliations:** ^1^ Nano and Bioelectrochemistry Research Laboratory CO2 and Green Technologies Research Centre Vellore Institute of Technology University Vellore 632 014 Tamil Nadu India; ^2^ Department of Chemistry School of Advanced Sciences Vellore Institute of Technology University Vellore 632 014 Tamil Nadu India

**Keywords:** 6‐hydroxy flavone, electrocatalysis, phenylhydrazine oxidation, redox‐active molecular catalyst, rotating disc electrode

## Abstract

The development of simple and rapid methods for preparing redox‐active molecular catalyst‐functionalized carbon electrodes for electrocatalytic applications is a significant research area. 6‐Hydroxyflavone (HFLA), a naturally occurring flavonoid with known anxiolytic properties, also acts as a noncompetitive inhibitor of cytochrome. This study focuses on the in situ functionalization of multiwalled carbon nanotubes (MWCNTs) with redox‐active HFLA, resulting in a modified electrode denoted as GCE/MWCNT@HFLA‐Redox, where HFLA‐Redox represents the redox‐active product of HFLA. The constructed‐modified electrode exhibits a well‐defined and stable surface‐confined redox response at E° of 0.55 V versus Ag/AgCl, with a surface excess of 6.26 × 10^−9^ mol cm^−2^ in a pH 2 KCl‐HCl solution. The modified electrode is characterized by Fourier transform infrared, Raman, UV–vis, field‐emission scanning electron microscopy, high‐resolution mass spectrometry (organic extract), and control electrochemical studies. This GCE/MWCNT@HFLA‐Redox electrode selectively oxidizes phenylhydrazine (PhHyd) in a pH 2 KCl‐HCl solution. A screen‐printed‐modified electrode facilitates highly selective electrocatalytic oxidation of PhHyd via amperometric *i*–*t* measurements and batch injection analysis, without interference from hydrazine or other common electroactive species. This method exhibits an excellent linear calibration curve (200 nM to 2 μM), demonstrating a high‐current sensitivity of 0.413 μA μM^−1^ cm^−2^ and a detection limit of 7 nM (signal‐to‐noise ratio of 3).

## Graphical abstract

This study presents a novel, rapid method for in situ functionalization of carbon electrodes using redox‐active form of the 6‐hydroxyflavone. The resulting multiwalled carbon nanotubes with redox‐active HFLA (MWCNT@HFLA‐Redox) electrode enables sensitive, selective detection of toxic phenylhydrazine, with excellent performance (limit of detection: 7 nM), stability, and eco‐compatibility, offering a cost‐effective alternative to conventional sensing techniques.

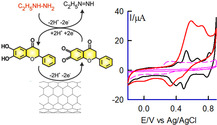

## 1 Introduction

Flavonoids, a widespread subclass of polyphenolic compounds, are abundant secondary metabolites found throughout the plant kingdom.^[^ ^1^ ^]^ Commonly present in fruits, vegetables, and beverages like tea, coffee, chocolate, and red wine, they contribute significantly to a healthy diet. These compounds have attracted considerable interest due to their potential health benefits. Hydroxylated flavones and their derivatives, naturally occurring with diverse pharmaceutical and biological applications, exhibit anti‐inflammatory,^[^ ^2^ ^]^ anticancer,^[^ ^3^ ^]^ antioxidant,^[^ ^4^, ^5^ ^]^ antidiabetic,^[^ ^6^ ^]^ and antimicrobial properties, thus contributing to the prevention of chronic diseases such as cancer, heart disease, and stroke.^[^ ^7^ ^]^ Flavonoids are recognized for their electroactive nature due to the presence of phenolic hydroxyl groups, which allow them to participate in electron‐transfer reactions and reduction processes due to their conjugated π‐electron systems and catalytic activity.^[^ ^8^, ^9^, ^10^ ^]^ Meanwhile, our group introduced a new electrochemical approach for in situ electrochemical transformation of phenol to surface‐confined hydroquinone moiety on multiwalled carbon nanotube (MWCNT) surface via phenoxy‐radical intermediate species formation.^[^ ^11^, ^12^ ^]^ To our knowledge, no specific electrochemical studies on HFLA have been reported. Given HFLA's unique properties, we conducted a systematic study to synthesize a redox‐active HFLA‐modified multiwalled carbon nanotube composite, MWCNT@HFLA‐Redox, HFLA‐Redox = Redox active quinone derivative of HFLA. We observed an in situ electrochemical oxidation of the phenolic site of HFLA on the MWCNT surface, resulting in its surface confinement as a redox‐active 1,2‐dihydroxy derivative. This synthesis method avoids conventional, time‐consuming techniques. Furthermore, we used biodegradable screen‐printed electrodes (SPEs) for enhanced stability and environmental compatibility. In this work, we observed a distinct, surface‐confined redox peak for HFLA at an apparent standard electrode potential (E^0^) of 0.55 V versus Ag/AgCl in a pH 2 KCl‐HCl solution. This peak serves as a qualitative and quantitative marker for HFLA analysis. We employed a range of characterization techniques, including physicochemical, spectrochemical, and electrochemical methods, to confirm the successful modification of the electrode. Phenylhydrazine (PhHyd) is a hazardous organic pollutant characterized by its toxic, carcinogenic, and corrosive properties. It finds widespread use across various industries, including the production of rocket propellants, agricultural pesticides, dyes, gas generators, explosives, chemical blowing agents, pharmaceutical intermediates, and photographic chemicals.^[^ ^13^, ^14^, ^15^ ^]^ While PhHyd offers valuable industrial applications, its inherent toxicity necessitates stringent handling procedures and effective decomposition strategies to mitigate its environmental impact. The oxidative decomposition of PhHyd^[^ ^16^, ^17^ ^]^ and its sensitive detection have been extensively documented in the literature.^[^ ^18^, ^19^ ^]^ Notably, established techniques for PhHyd detection include chemiluminescence,^[^ ^20^ ^]^ chromatography,^[^ ^21^, ^22^, ^23^ ^]^ and spectrophotometry.^[^ ^24^, ^25^ ^]^ However, these standard analytical methods often suffer from drawbacks such as lengthy analysis times, high costs, and the need for complex derivatization procedures. In contrast, electroanalytical techniques offer distinct advantages, including direct analysis, high sensitivity, simplicity, reliable selectivity, rapid response, and cost‐effectiveness. For the electrochemical oxidation of PhHyd, chemically modified electrodes incorporating transition metal‐based nanoparticles have demonstrated promising results. Examples include,^[^ ^26^, ^27^, ^28^ ^]^ Ag/Al‐ZnO,^[^ ^29^, ^30^ ^]^ GCE/NiCoNP,^[^ ^17^ ^]^ and a carbon paste electrode modified with TiO_2_ nanoparticles and 2,2′[1,2‐butanediylbis (nitriloethylidyne)]‐bis‐hydroquinone (BHTCPE).^[^ ^31^ ^]^ Nevertheless, the time‐consuming preparation procedures and the high cost of precious metal components present significant challenges. In this study, MWCNT@HFLA‐Redox is introduced as a high‐performance electrocatalyst for the oxidation and sensing of PhHyd in aqueous solutions. Electrochemical impedance spectroscopy (EIS) and amperometric i–t studies were conducted to investigate the effective electrochemical oxidation of PhHyd. This novel system exhibited a current sensitivity of 0.413 μA μM^−1^ cm^−2^ and a detection limit of 7 nM. The electroanalytical performance of this new system surpasses that of previously reported methods (**Table** **1**).^[^ ^17^, ^29^, ^30^, ^31^, ^32^, ^33^, ^34^, ^35^, ^36^ ^]^ Building upon these findings, it is evident that the developed system demonstrates exceptional selectivity, high stability, and innovation in the direct electrocatalytic oxidation of PhHyd. To validate the concept, the MWCNT@HFLA‐Redox‐modified SPE was employed as an electrochemical detector for batch injection analysis (BIA) of PhHyd in various environmental real samples.

**Table 1:** Comparison of the electroanalytical parameters for PhHyd detection by present work with the reported literature data.

S. No.	Modified system	Technique	Current sensitivity [μA μM^−1^ cm^−2^]	LR [μM]	DL [μM]	Ref
1	Ag/Al‐ZnO	CV	64.77	313‐2500	313	29
2	GCE/NiCoNP	CV	0.086	500‐12,000	266	17
3	AgE/Al‐ZnO NPs	I‐V	0.014	10‐50,000	1.21	30
4	BHTCPE	DPV	0.069	2‐1000	0.75	31
5	Modified CNT tube/Ferrocene	Amp. i‐t	0.039	0.85‐700	0.6	32
6	Modified carbon paste/FMC	DPV	0.038	0.8‐700	0.42	33
7	Modified carbon nanotube paste electrode (MCNPE)	Chrono‐ amperometry	0.030	0.6‐900	0.131	34
8	MBIZBr/CoFe_2_O_4_ NPs/CPE	SWV	0.340	0.03‐750	0.007	35
9	[Ru(II)tpyphCl]/MWCNTs/nafion	CV	0.020	5‐200	0.15	36
10	MWCNT@HFLA‐Redox	BIA	0.413	0.2‐2	0.007	This work

**Note:** LR: linear range; DL: detection limit; FMC: ferrocene monocarboxylic acid; BHTCPE: carbon paste electrode with TiO_2_ nanoparticles and a derivative of hydroquinone (2,2′[1,2 butanediyl bis(nitriloethylidyne)]‐bis‐hydroquinone, BH); MBIZBr: 1‐methyl‐3‐butylimidazolium bromide.

## 2 Experimental Section

### 2.1 Chemicals and Reagents

6‐Hydroxyflavone (98% purity, TCI, Japan), MWCNTs (≥98 % purity on a carbon basis, 10–11 nm outer diameter, 4–5 nm inner diameter, 3–6 μm length), COOH‐functionalized MWCNTs (f‐MWCNTs, ≈8% functionalization with COOH groups, ≈9.5 nm diameter, ≈1.5 μm length), carbon black (CB, N330 grade, a gift from Phillips Carbon Black Ltd., Kochi, India), carbon nanofibers (CNFs, ≈99% purity, 100 nm × 2–200 μm, Aldrich, USA), graphitic nanopowder (GNP, ≈98% purity), graphene oxide (GO, >80% purity, 0.6–1.2 nm thickness, 0.5–2.0 μm flake size), and graphitized mesoporous carbon (GMC, 99.95% purity, <50 nm particle size) were used as received and stored appropriately. Potassium ferrocyanide (K_4_[Fe(CN)_6_]) and potassium ferricyanide (K_3_[Fe(CN)_6_]) were obtained from Sigma‐Aldrich, USA. A 0.1 M supporting electrolyte (pH 2) was prepared by mixing KCl and HCl and used throughout the study. All aqueous solutions were prepared using deionized, double‐distilled, alkaline permanganate‐treated water. The pH of solutions was adjusted between 2 and 13 using 0.1 M H_3_PO_4_ or 0.1 M NaOH. Phosphate buffer solutions (0.1 M PBS) were prepared by mixing and diluting standard solutions of Na_2_HPO_4_ and NaH_2_PO_4_. Phenylhydrazine (reagent grade, SDFCL, Mumbai) was used as received, and 1 mM stock solutions were prepared for all electrochemical experiments. Dissolved oxygen did not affect the electrochemical reaction; therefore, N_2_ purging was unnecessary. **Caution!** Phenylhydrazine is toxic and a suspected carcinogen; handle it with appropriate safety precautions.

### 2.2 Instrumentation

Electrochemical experiments, including hydrodynamic amperometric *i–t* measurements, were performed using a CHI 440b bipotentiostat workstation (Austin, USA). The electrochemical setup consisted of a reference electrode (Ag/AgCl with 3 M KCl), a counter electrode (platinum wire), and a working electrode (glassy carbon electrode, GCE; 3 mm diameter, 0.0707 cm^2^ surface area) housed in a 10 mL electrochemical cell. The GCE (BAS Inc., Japan) was polished before use with a polishing kit (Bioanalytical Systems, USA). Fourier transform infrared (FT‐IR) spectral analysis was performed using a Shimadzu Affinity‐1 instrument (Japan). Raman spectroscopy was conducted with an i‐Raman Plus 532 H apparatus (Switzerland) equipped with a 532 nm laser. UV–vis spectroscopy measurements were carried out using a Jasco V‐700 spectrometer (Japan) with a transparent ethanolic extract. Surface morphology was studied using field‐emission scanning electron microscopy (FESEM) on an FEI Quanta SEM instrument. High‐resolution mass spectrometry (HRMS) analysis was performed using an Avion compact mass spectrometer (CMS expression). For HRMS analysis, the modified electrode was sonicated in 1 mL of methanol, and the resulting solution was filtered through a 0.2 μM syringe filter. Rotating disc electrode (RDE) experiments were conducted using a glassy carbon disc/gold electrode with an RDE‐2 Rotating Ring Disk Electrode instrument (BASi, USA). Finally, highly sensitive BIA was performed using a programmable Dropsens pipette (20 μL), a BIASPE10 sample injection apparatus, and an Autolab workstation (Metrohm, Switzerland).

### 2.3 Preparation of MWCNT@HFLA‐Redox‐Modified Electrode

**Scheme 1:**
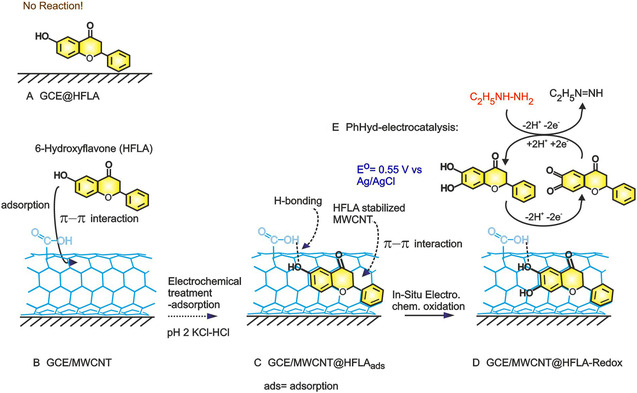
A) Schematic representation of the GCE/HFLA, B,C) HFLA adsorbed GCE/MWCNT via *π*–*π* interaction, D) GCE/MWCNT@HFLA‐Redox, and E) and its mediated oxidation of PhHyd reaction.

≈2 mg of MWCNTs were dispersed in 500 μL of ethanol and sonicated for 15 min. A glassy carbon electrode (GCE, 3 mm diameter) was mechanically cleaned by polishing with 0.5 μM alumina (Al_2_O_3_) powder, rinsed with double‐distilled water, and then sonicated for 5 min to ensure thorough cleaning. The GCE was electrochemically pretreated using cyclic voltammetry (CV) for 10 cycles at a scan rate of 50 mV s^−1^ within a potential window of −0.2 to + 1.0 V versus Ag/AgCl in pH 7 PBS. To fabricate the GCE/MWCNT@HFLA‐modified electrode, 5 μL of the ethanol‐dispersed MWCNT solution was drop‐cast onto the bare GCE and air‐dried for 3 min at room temperature (**Scheme** **1**A). Ten consecutive CV cycles were performed in a pH 2 KCl‐HCl solution within a potential range of −0.1 to + 0.9 V. Subsequently, 4 μL of a dispersion containing 1 mg of HFLA in 300 μL of absolute ethanol (Scheme 1B and C) was coated onto the GCE/MWCNT electrode. The electrode was air‐dried and then subjected to another ten CV cycles in the same pH 2 KCl‐HCl solution at a scan rate of 50 mV s^−1^ within the same potential range. The resulting modified electrode was designated GCE/MWCNT@HFLA‐Redox (Scheme 1D).

## 3 Results and Discussion

### 3.1 Electrochemical Performance of HFLA on the MWCNT‐Functionalized Electrode

**Figure 1:**
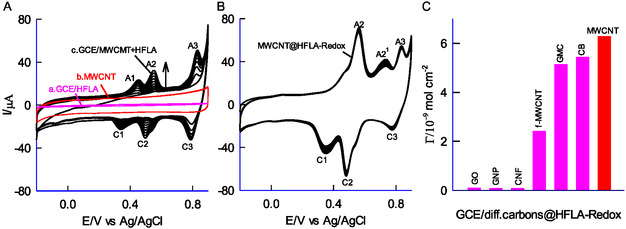
A) Ten continuous CV responses of GCE with HFLA (a), MWCNT (b), and GCE/MWCNT + HFLA (c) in pH 2 KCl‐HCl buffer solution at *v *= 50 mV s^−1^. B) Ten continuous CV responses of MWCNT@HFLA‐Redox in a blank pH 2 KCl‐HCl solution (optimal electrode). C) Comparative plot of surface excess vs various carbons to prepare CME in pH 2 KCl‐HCl solution.

A preliminary CV experiment was conducted using both unmodified glassy carbon electrodes with HFLA and with multiwalled carbon nanotubes (GCE/MWCNT) in a deoxygenated solution of pH 2 KCl + HCl. As shown in **Figure** **1**A (curves a and b), no faradaic activity was observed within the examined potential range. This indicates that HFLA exhibits no electrochemical activity on the solid electrode. Subsequently, the GCE/MWCNT electrode was modified with HFLA, creating a chemically modified electrode (CME), designated GCE/MWCNT@HFLA‐Redox. The CV response of this CME (Figure 1A, curve c) showed three distinct redox peak pairs (A1/C1, A2/C2, and A3/C3) across twenty consecutive cycles. The formal potentials (E°') were determined to be 0.446 ± 0.020 V for A1/C1, 0.551 ± 0.010 V for A2/C2, and 0.820 ± 0.025 V (vs Ag/AgCl). The CME was then rinsed with deionized water, and CV was performed again in a blank pH 2 KCl‐HCl solution (Figure 1B). The A2/C2 redox peak demonstrated excellent stability, with no significant changes in peak current or potential. This stability is attributed to efficient electron transfer between the surface‐confined HFLA redox species (HFLA‐Redox) and the MWCNTs (Figure 1B), in contrast to the lack of activity observed with HFLA on the bare GCE (Figure 1A, curve a). The surface excess value calculated for the A2 peak was 6.26 × 10^−9^ mol cm^−2^, with a relative standard deviation (RSD) of 3.2% across ten consecutive CV cycles, confirming the excellent stability and reproducibility of the CME.

To investigate the influence of carbon nanomaterials on electrochemical redox reactions, a selection of carbon materials – namely, GO, graphene nanopowder (GNP), CNFs, oxygen‐functionalized carbon nanotubes (f‐CNT), GMC, CB, and MWCNT, were employed (Figure 1C and Figure S1, Supporting Information). Figure 1C depicts the surface excess (Γ_HFLA‐Redox_) as a function of different carbon nanomaterials (interactions between HFLA and graphitic sites, including *π*‐*π* stacking and surface effects on redox formation). The ranking of these materials based on HFLA formation is as follows: MWCNT > CB > GMC > f‐MWCNT > CNF > GNP > GO. Among them, MWCNT exhibits the highest Γ_HFLA‐Redox_ value, emphasizing that its edge‐plane sites,^[^ ^37^ ^]^ provide the most favorable environment for the immobilization of HFLA‐Redox in this work. The surface reactive site concentration was optimized to ≈13.33 μg of HFLA on the MWCNT surface by adjusting the amounts through deposition optimization experiments (Figure S2, Supporting Information).

### 3.2 Electrochemical Analysis of the MWCNT@HFLA‐Redox System

**Figure 2:**
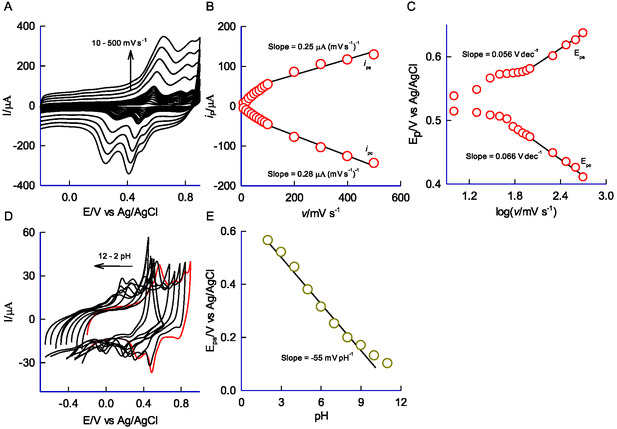
Effect of scan rate (10–500 mV s^−1^) on CV response of A) MWCNT@HFLA‐Redox in pH 2 KCl‐HCl. B) A plot of anodic (*i*_pa_) and cathodic peak (*i*_pc_) currents versus scan rate of CME and a respective plot of C) E_p_ versus log(*v*). D) Effect of pH (2–12) on the CV response of MWCNT@HFLA‐Redox at a fixed scan rate of 50 mV s^−1^. E) Plot of E_pa_ versus pH.

To investigate the electron‐transfer behavior, the GCE/MWCNT@HFLA‐Redox electrode was subjected to a scan rate ranging from 100 to 500 mV s^−1^ in a blank pH 2 KCl−HCl electrolyte solution. As depicted in **Figure** **2**A, a consistent variation in anodic (*i*_pa_) and cathodic (*i*_pc_) redox peak currents was observed concerning scan rates. The linear plot of *i*_pa_ and *i*_pc_ versus *v* for the A2/C2 redox peak (Figure 2B) originating from zero, suggests the surface‐confined electron transfer behavior of the redox system.^[^ ^38^ ^]^ The anodic (*i*_pa_) to cathodic (*i*_pc_) peak current ratio is ≈1 at low scan rates (10–100 mV s^−1^) and rises to around 1.5 at high scan rates (1,000 mV s^−1^), suggesting a dual kinetic behavior (adsorption as well as mixed diffusion‐controlled electron transfer) on the modified electrode. At high scan rates, it is likely to experience an electron‐hopping mechanism,^[^ ^39^, ^40^, ^41^, ^42^, ^43^ ^]^ where electrons jump from one redox center to an adjacent site, facilitated by a diffusion‐controlled electron transfer process. The Laviron model for electrode kinetics^[^ ^38^, ^44^ ^]^ was applied to determine the transfer coefficient (*α*) and the heterogeneous electron transfer rate constant (*k*_s_).

(1) $$S1_{a}/S1_{c}=\alpha /\left(1-\alpha \right)$$

(2) $$\log(k_{s})=\alpha \log(1-\alpha )+(1-\alpha )\log\alpha -\log[(\text{RT}/\{\text{nF}(v/{\text{Vs}}^{-1}\})]-\alpha (1-\alpha )\text{nF}\cdot E_{p}/2.3\text{RT})$$

In the above context, Sl_a_ and Sl_c_ represent the slopes of the anodic (E_pa_) and cathodic (E_pc_) plots versus log(*v*/mV dec^−1^), where *v* denotes the scan rate (mV s^−1^), ΔEp refers to the peak‐to‐peak separation (mV), n represents the number of electrons transferred, and F is Faraday's constant (C mol^−1^). All other symbols carry distinct meanings relevant to this context. A plot of E_pa_ and E_pc_ versus log*v* showing two distinct linear responses for both anodic and cathodic processes (Figure 2C), reflecting changes in the kinetics. The anodic (Sl_a_) and cathodic (Sl_c_) slope values are determined to be 56 mV dec^−1^ and 66 mV dec^−1^, respectively. From Equations (1) and (2), the derived *α* and *k*_*s*_ values are 0.46 and 0.219 s^−1^. The observed transfer coefficient value is nearer to the ideal value of 0.5 indicating the symmetry electron‐transfer function of the modified electrode. Further, the modified electrode was analyzed in pH solutions ranging from 2 to 12 at a constant scan rate of 50 mV s^−1^ (Figure 2D). A consistent shift in the redox peaks with varying pH was observed, suggesting that the redox couple operates via proton‐coupled electron transfer mechanism. Figure 2E presents a plot of E_pa_ versus pH, yielding a linear line. The obtained slope of −55 mV pH^−1^ is closely aligned with the ideal value of −58 mV pH^−1^ indicating a reversible electron transfer associated with a proton‐coupled under Nernstian behavior. Identifying the specific active species adsorbed on the GCE/MWCNT@HFLA‐Redox electrode poses a significant challenge. Determining the adsorbed organic species at the intricate electrode‐electrolyte interface is especially difficult due to the extremely small quantities involved, often in the nanogram range. To overcome this challenge and elucidate the structure of the adsorbed organic species, we utilized carefully designed electrochemical and physicochemical characterization techniques. The findings from these analyses are detailed in the following sections.

### 3.3 Physicochemical Characterization of MWCNT@HFLA‐Redox

**Figure 3:**
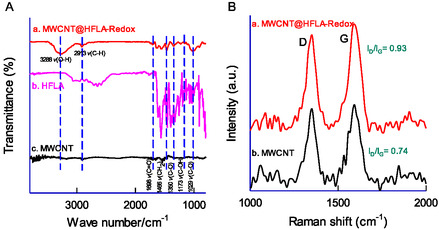
A) FT‐IR responses of MWCNT@HFLA‐Redox (a), HFLA (b), and MWCNT (c). B) Raman spectroscopic responses of MWCNT@HFLA‐Redox (a), and MWCNT (b).

Curves A, B, and C in **Figure** **3**A present the comparative FT‐IR responses of MWCNT@HFLA‐Redox, HFLA, and MWCNT, highlighting characteristic stretching signals for various functional groups. Distinct IR peaks were observed at 3288 cm^−1^ (*v*:*O*‐H), 2913 cm^−1^ (*v*:C‐H), 1698 cm^−1^ (*v*:C=O), 1466 cm^−1^ (*v*:CH=CH), and 1173 cm^−1^ (*v*:C‐OH) and compared with control systems like HFLA and MWCNT. The carbonyl (*v*:C=O) functional group exhibited a notable shift in its IR peaks from 1718 cm^−1^ to 1698 cm^−1^ between HFLA and MWCNT@HFLA‐Redox further indicating that the immobilized oxygenated organic molecular system is confined to the surface (potentially accounting for the observed A2/C2 redox peak). Plausibly, a 1,2‐dihydroxy benzene‐like derivative of HFLA will be formed on the surface and stabilized by the MWCNT matrix. In parallel, the *π*‐*π* interaction between the aromatic electrons of HFLA and sp^2^ carbons of MWCNT promotes surface bonding, confirmed by Raman spectroscopy. Raman analysis distinguishes sp^3^ (defective/disordered) and sp^2^ (ordered) bonding,^[^ ^45^ ^]^ with the D and G bands at 1345 and 1586 cm^−1^, respectively (Figure 3B). The I_D_/I_G_ ratio, a sensitive index of molecular structure, reflecting graphitic disorder, is 0.74 for MWCNT and 0.93 for MWCNT@HFLA‐Redox, indicating an enormous increase in the D‐band intensity, suggesting the introduction of sp^3^ molecule units from HFLA‐Redox onto the MWCNT surface. This signifies the formation of a hydroquinone molecule, during the electrochemical reaction. Further to strengthen the result, the UV–vis spectroscopy was performed with MWCNT@HFLA‐Redox (ethanoic extract) and HFLA (Figure S3, Supporting Information) obtained by sonicating in 1 mL of ethanol and then micro‐syringe (0.2 μM) filtration. The UV–visible spectrum of pristine HFLA exhibits a *λ*_max_ at 317 nm, in comparison, the MWCNT@HFLA‐Redox sample reveals the peak at *λ*_max_ = 303 nm. The observed absorption wavelength shift reflects structural modifications through an in situ electrochemical process, pointing to the formation of a quinone‐like product. This outcome confirms the successful synthesis of a novel organic product (HFLA‐Redox) in the work.

**Figure 4:**
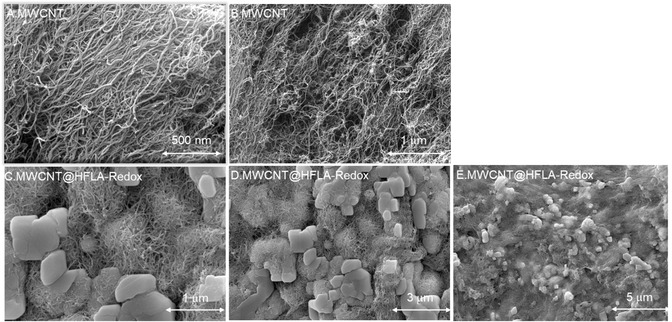
A,B) Typical FE‐SEM images of MWCNT and C,D,E) MWCNT@HFLA‐Redox.

The HRMS response of the methanolic solution extract, shown in Figure S4, Supporting Information displays a prominent molecular ion peak at *m/z* = 256.07. This peak corresponds to the isolated HFLA‐quinone species, with an observed *m/z* of 256.28, confirming the anticipated result of 1,2‐dihydrobenzene derivative of HFLA as redox species. **Figure** **4**A,B illustrates the FESEM images of MWCNT, revealing its characteristic twisted morphology. Meanwhile, Figure 4C,D,E displays MWCNT@HFLA‐Redox, where the formation of microcrystalline structures and the aggregation of carbon nanotubes indicate the effective attachment of electroactive species onto the MWCNT surface, potentially driven *π*‐stacking interactions between the organic molecule and the underlying graphitic unit (Scheme 1). Based on the observation it is accounted that the A2/C2 peak is HFLA‐Redox and other minor peaks (A3/C3) are due to adsorption of the redox‐active sites on energetically different sites of MWCNT (basal planes and some metal impurity trapped sites).

**Figure 5:**
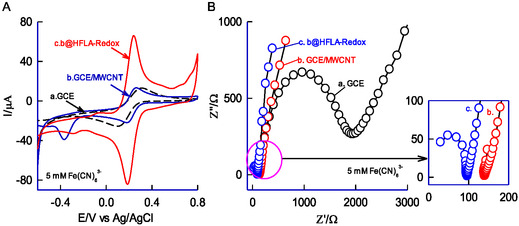
A) CV responses of GCE (a), GCE/MWCNT (b), and GCE/MWCNT@HFLA‐Redox (c) with 5 mM Fe(CN)_6_^3−^ dissolved in 0.1M KCl at v = 50 mV s^−1^. B) Comparative electrochemical impedance spectroscopic response of GCE (a), GCE/MWCNT (b), and GCE/MWCNT@HFLA‐Redox (c) in 5 mM Fe(CN)_6_^3−^ containing 0.1M KCl electrolyte at an applied potential of 480 mV versus Ag/AgCl. Inset is the enlarged view of a portion.

The surface properties of the CME were compared with GCE and GCE/MWCNT using the FeIII/II(CN)6^3−^ ^/4−^ redox couple in a 0.1 M KCl solution and was displayed in **Figure** **5**A. All three systems exhibited similar qualitative voltammetric behavior; however, their peak current values varied depending on the electrode modification, ranking as follows: GCE/MWCNT@HFLA‐Redox > GCE/MWCNT > GCE. The GCE/MWCNT@HFLA‐Redox displayed the highest peak current, highlighting its superior electrical performance. This improvement is likely due to the presence of electroactive organic species in the matrix, which may have formed during the electrochemical modification process, responsible for increased electrical conductivity of the film. EIS is a reliable technique for evaluating the interfacial characteristics of modified electrode surfaces. The measurements were carried out within a frequency range of 1 Hz to 1 MHz using an AC potential of 10 mV, and the results are presented as Nyquist plots in Figure 5B (curves a‐c). The impedance data for GCE and GCE/MWCNT@HFLA‐Redox reveal two key components: a linear segment linked to the diffusion‐limited process and a semicircular segment corresponding to the electron transfer process. The diameter of the semicircular region reflects the charge transfer resistance (R_CT_). The R_CT_ values were found to be 1876 Ω for GCE, 140 Ω for GCE/MWCNT, and 93 Ω for GCE/MWCNT@HFLA‐Redox. The decrease in R_CT_ for GCE/MWCNT@HFLA‐Redox demonstrates enhanced electrical conductivity, resulting from the inclusion of HFLA molecular species. The semicircular portion of the EIS data was fitted using the Randles circuit model, R_s_[R_ct_C_dl_], wherein, R_s_ = solution phase resistance, R_ct_ = charge‐transfer resistance, and C_dl_ = electrical double layer capacitance.

### 3.4 Electrocatalytic Oxidation of PhHyd on MWCNT@HFLA‐Redox

**Figure 6:**
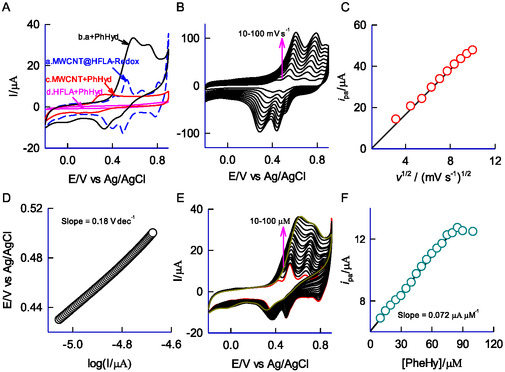
A) Comparative CV responses of MWCNT@HFLA‐Redox (b), MWCNT (c), and HFLA (d) with 100 μM of PhHyd in pH 2 HCl‐KCl at *v* = 10 mV s^−1^. Curve (a) is a control CV of GCE/MWCNT@HFLA without PhHyd. B) CV response of GCE/MWCNT@HFLA‐Redox with 100 μM PhHyd at different scan rates. C) A plot of anodic peak current (*i*_pa_) versus *v* ^1/2^ for the PhHyd oxidation reaction on GCE/MWCNT@HFLA‐Redox in pH 2 KCl‐HCl. D) Tafel plot for 100 μM PhHyd oxidation at GCE/MWCNT@HFLA‐Redox in pH 2 KCl‐HCl. The rising portion of the current–voltage curve recorded at *v* = 10 mV s^−1^ (Figure 7 E was used). E) CV responses of GCE/MWCNT@HFLA‐Redox without and with various concentrations of PhHyd (10‐100 μM) in pH 2 HCl‐KCl at *v* = 10 mV s^−1^. F) Plot of *i*_pa_ versus concentration of PhHyd in μM.

The comparative CV responses of MWCNT, HFLA, and MWCNT@HFLA‐Redox for the electrochemical oxidation of 100 μM PhHyd in pH 2 KCl‐HCl display a distinct oxidation peak at E_pa_ = 0.55 V versus Ag/AgCl, where the A2/C2 redox peak appears and are presented in **Figure** **6**A. The CV response of PhHyd on GCE/MWCNT@HFLA‐Redox (Figure 6A, curve b) shows roughly 200‐fold enhancement in the oxidation peak than compared to control electrodes (Figure 6A, curve a, c, and d) indicating the efficient electrocatalytic activity of PhHyd. It is noted that MWCNT exhibited a weak and unstable oxidation peak at a lower potential of 0.35 V (Figure 6, curve c). This is likely due to the limited mediated activity of some trapped metals (Fe, Ni, and Co) within the MWCNT.^[^ ^41^ ^]^ However, the exact details of this phenomenon remain unknown. Figure 6B depicts the effect of scan rate (10–100 mV s^−1^) on the CV response of GCE/MWCNT@HFLA‐Redox with 100 μM PhHyd in pH 2 KCl‐HCl. A clear increase in the oxidation peak current is observed as the scan rate is raised. In Figure 6C, the plot of oxidation peak current (*i*_pa_) against the square root of scan rate (*v* ^1/2^) shows a linear trend beginning from the origin. This suggests that the electrochemical oxidation of PhHyd is governed by a diffusion‐controlled process. In continuation, the anodic Tafel slope (Figure 6D), which is a key kinetic parameter used to evaluate the reaction rate in electrochemical processes, was calculated to be 180 mV dec^−1^ from the CV data for the electrocatalytic oxidation response, using the E versus logI plot. The transfer coefficient for PhHyd oxidation was estimated to be 0.33, which is slightly lower than the ideal value of 0.5. This discrepancy suggests a minor asymmetry of the electron‐transfer energy barrier and is responsible for the irreversible electron‐transfer reaction. Figure 6E depicts the effect of PhHyd concentration (10–100 μM) on the CV response of MWCNT@HFLA‐Redox in pH 2 KCl‐HCl. The oxidation peak current steadily increased with rising PhHyd concentration, up to 90 μM, beyond which the response reached a plateau. A calibration curve for PhHyd was constructed based on the CV of the electrocatalytic oxidation reaction by plotting peak current values against concentration, as shown in Figure 6F. This observation exemplifies Michaelis–Menten kinetics, specifically the substrate–analyte binding kinetics observed in this oxidation system.^[^ ^46^ ^]^ The plot exhibited linearity in the range of 10–90 μM, with a current sensitivity of 0.072 μA μM^−1^ and regression coefficient of 0.9826.

### 3.5 Rotating Disc Electrode Analysis of PhHyd

**Figure 7:**
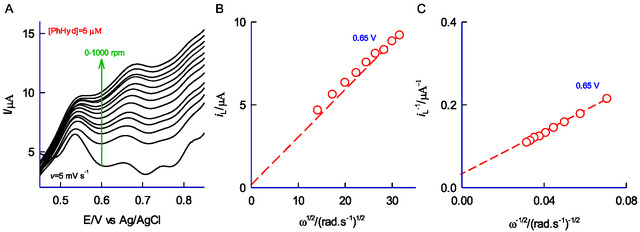
A) RDE responses for the oxidation of 5 μM PhHyd on GCE/MWCNT@HFLA at different rpms (0‐1000) at *v* = 5 mV s^−1^. B) Levich plot and C) Koutecky–Levich plot.

A RDE, a type of hydrodynamic working electrode system, was used to evaluate the reaction rate constant.^[^ ^47^ ^]^ The GCE‐disc portion was modified with HFLA‐CME. The above electrode was operated at rotational speeds ranging from 0 to 1,000 rpms, with a fixed scan rate of 5 mV s^−1^, as displayed in **Figure** **7**A. A significant increase in the oxidation peak current was detected with increasing rpm. Figure 7B depicts a plot of limiting current, *i*_L_ against the square root of the angular velocity (ω^1/2^) at 0.65 V versus Ag/AgCl. The linear curve starting from the origin indicates that the RDE behavior for the PhHyd complies with the Levich equation, as expressed below.

(3) $$i_{L}=\left[0.62{\text{nFAD}}^{2/3}v^{-1/6}C_{\text{PhHyd}}\right]\omega^{1/2}\;\left(3\right)$$

wherein *i*_L_ refers to the hydrodynamic current measured at the limiting current. The observed linear relationship reinforces the diffusion‐controlled electron transfer mechanism of the PhHyd (Figure 2B). To calculate the kinetic parameters, the Koutecky–Levich (KL) equation is applied.

(4) $$1/i_{L}=1/i_{k}+1/0.62{\text{nFAD}}^{2/3}v^{-1/6}C_{\text{PhHyd}}{\text{ ω}}^{1/2}$$

(5) $$i_{k}=n\text{FA}k_{h}C_{\text{PhHyd}}\text{Γ }$$

In this context, *k*_h_ denotes the heterogeneous rate constant of CME, *i*_k_ represents the kinetic current, and other symbols have their respective meanings.^[^ ^48^ ^]^ The formation of a quinone structure is responsible for the obstruction in the electron‐transfer behavior of HFLA‐Redox system. A plot of *i*_L_^−1^ versus ω^−1/2^ observed a linear trend, and the corresponding *i*_k_ value (Eq. 5) was calculated from the intercept (Figure 7C). The calculated *k*_h_ value is 18.2 s^−1^, highlighting fast electron‐transfer reaction for the oxidation reaction.

### 3.6 Amperometric *i*–*t* Analysis of PhHyd on MWCNT@HFLA‐Redox

**Figure 8:**
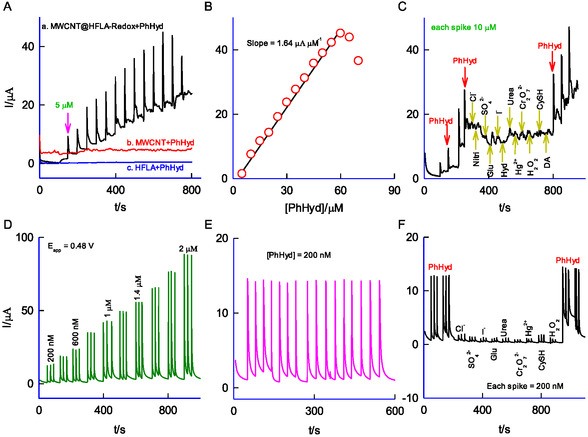
A) Comparative amperometric *i–t* responses of GCE/MWCNT@HFLA‐Redox (a), GCE/MWCNT (b), and GCE/HFLA (c) for successive spikes of 5 μM PhHyd at E_app_ = 0.48 V versus AgCl in 10 mL pH 2 KCl‐HCl buffer solution (hydrodynamic condition). B) Typical calibration plot and C) amperometric *i–t* response of the GCE/MWCNT@HFLA‐Redox with successive additions of 5 μM of different interfering chemicals, PhHyd, Cl^−^, nitrite, SO_4_^2−^, Glu, I^−^, Hyd, urea, Hg^2+^, Cr_2_O_7_^2−^, H_2_O_2_, CySH, and DA. D) BIA response of GCE/MWCNT@HFLA‐Redox for increasing concentration of PhHyd (200 nM – 2 μM), E) Repeatability test: sixteen repeated spikes of 200 nM PhHyd and F) interference compounds effect on the detection of PhHyd. BIA parameters: E_app_ = 0.48 V versus Ag/AgCl; spikes = 20 μL; buffer solution = pH 2 KCl‐HCl.

This technique is ideal for conducting real‐time monitoring of PhHyd samples. The typical amperometric response of MWCNT@HFLA‐Redox to successive spikes of 5 μM PhHyd in pH 2 KCl‐HCl at an applied potential of 0.48 V versus Ag/AgCl under hydrodynamic condition is shown in **Figure** **8**A. A consistent step‐like increase in the current value was observed. The PhHyd calibration curve showed linearity from 5 to 60 μM, with a current sensitivity and regression coefficient of 1.64 μA μM^−1^, and 0.9558 respectively. Ten consecutive amperometric measurements of 5 μM PhHyd resulted in a RSD of 4.8%, with the detection limit calculated to be 3.7 μM (S/N = 3), emphasizing the strong analytical applicability of this new system. In contrast, the unmodified MWCNT and HFLA with PhHyd exhibited a very poor response (Figure 8A, curves b and c). An interference study was performed on the working electrode by assessing the current response following the addition of PhHyd, chloride (Cl^−^), nitrite (NO_2_^−^), sulfate (SO_4_^−2^), glucose (Glu), iodate (I^−^), hydrazine (Hyd), urea, Mercury (Hg^2+^), chromate (Cr_2_O_7_^2−^), hydrogen peroxide (H_2_O_2_), cysteine (CySH), and dopamine (DA) in pH 2 KCl‐HCl. As illustrated in Figure 8C, only PhHyd induced a significant current response, while the other electroactive species showed no such effect, highlighting the selective and specific electroanalytical capability of the MWCNT@HFLA‐Redox electrode for detecting PhHyd in this study.

### 3.7 BIA of PhHyd

**Figure 9:**
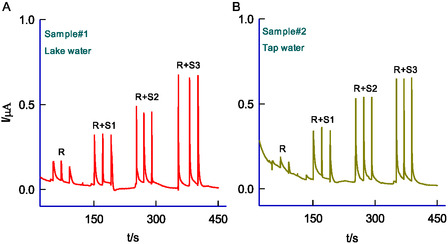
A) BIA response of GCE/MWCNT@HFLA‐Redox for real sample#1 (Lake water) and B) sample#2 (Tap water) by standard addition approach (standard [PhHyd] are, 200 nM – 600 nM). Note: Other BIA parameters are mentioned in the Figure 8.

To enable real‐time practical applications, this innovative approach was adopted for integration into a portable‐type BIA technique. This approach extends the capabilities of the amperometric *i*–*t* technique by incorporating a customized electrochemical detection system that utilizes a three‐in‐one SPE modified with MWCNT@HFLA‐Redox as the working electrode, a programmable micropipette for precise sample injection (20–100 μL), and a sample detection system with a capacity of 60–100 mL.^[^ ^49^ ^]^ Key analytical parameters, including the stirring speed of the BIA system, the volume of sample injection, and the applied electrochemical potential, were meticulously optimized before initiating the analysis. Figure 8D illustrates a typical BIA response of MWCNT@HFLA‐Redox to increasing PhHyd concentrations ranging from 200 nM to 2 μM, recorded at a bias potential of 0.48 V versus Ag/AgCl. The results exhibit a clear and systematic enhancement in sensing currents with increasing PhHyd levels. A linear calibration plot was observed within the concentration range of 200 nM to 2 μM, with a current sensitivity of 0.413 μA μM^−1^ cm^−2^ and a regression coefficient of 0.9899. The repeatability of the electrochemical detector was assessed using sixteen sequential injections of 200 nM PhHyd into the BIA cell (Figure 8E). This resulted in highly consistent and reproducible current signals, with a RSD of 3.9%, confirming the high repeatability and precision for electrochemical measurements under the given conditions. The limit of detection was determined to be 7 nM (3*σ*/slope), where σ is the standard deviation (9.65  × 10^−8^ μA) and the slope is derived from the linear regression of the calibration plot (4.13  × 10^−5^ μA/μM), under the specified analytical conditions and compared with previous results, summarized in Table 1, indicates that the newly modified system exhibits comparable low detection limits and showcases superior electrocatalytic properties for PhHyd detection. The potential interference from Cl^−^, SO_4_^2−^, I^−^, Glu, urea, Cr_2_O_7_^2−^, Hg^2+^, CySH, and H_2_O_2_ were performed (Figure 8F). The results revealed no significant interference, suggesting that these substances do not affect effective sensing and selectivity of PhHyd. As evidence of its feasibility, real sample analyses were conducted using lake water (**Figure** **9**A) and tap water (Figure 9B) via the standard addition method. The calculated recovery values, ≈ 101 ± 2 %, highlight the remarkable regeneration ability of the newly prepared sensor electrode.

## 4 Conclusion

In this study, a chemically modified electrode was fabricated by electrochemically oxidizing 6‐hydroxyflavone (HFLA) in situ on a MWCNT‐modified electrode. This resulted in a highly redox‐active molecular electrocatalyst, denoted as MWCNT@HFLA‐Redox, in a pH 2 KCl‐HCl solution. The modified electrode exhibited well‐defined redox peaks at E^o^ = 0.55 V (A2/C2) versus Ag/AgCl, indicating a surface‐confined electron transfer mechanism. The heterogeneous electron transfer rate constant, calculated using the Laviron equation, was determined to be 18.2 s^−1^. The presence of HFLA‐quinone‐like redox‐active species on the electrode surface was confirmed through various physicochemical characterization techniques, including FT‐IR, UV‐visible, Raman spectroscopy, FESEM, and HRMS, as well as electrochemical investigations. The electrocatalytic oxidation of phenylhydrazine (PhHyd) was examined using CV and RDE techniques. Key kinetic parameters, such as the transfer coefficient (α), the total number of electrons transferred (n), the Tafel slope (*b*_a_), and the heterogeneous electron transfer rate constant (*k*_h_), were determined. The amperometric *i*–*t* technique and BIA were employed to evaluate the electrocatalytic properties for PhHyd sensing. The results demonstrated high‐performance PhHyd oxidation with excellent selectivity, showing minimal interference from other electroactive species, including Cl^−^, SO_4_^2−^, I^−^, glucose, urea, Cr_2_O_7_^2−^, Hg^2+^, cysteine (CySH), and H_2_O_2_. Finally, the BIA technique, utilizing screen‐printed electrochemical technology, was successfully applied for real‐time PhHyd analysis in lake and tap water samples, achieving a recovery rate of 101 ± 2%.

## Conflict of Interest

The authors declare no conflict of interest.

## Supporting information

Supplementary Material

## Data Availability

The data that support the findings of this study are available on request from the corresponding author. The data are not publicly available due to privacy or ethical restrictions.
