# Supplementary material for: High‐Performance Oxidation and Nanomolar Detection of Phenylhydrazine Using a 6‐Hydroxyflavone‐Based Molecular Electrocatalyst Functionalized Multiwalled Carbon Nanotube in Batch Injection Analysis
Source: ChemistryOpen. 2025 Jun 9;14(11):e202500140. doi: 10.1002/open.202500140 (PMC12598829; doi:10.1002/open.202500140)
Supplement: Supplementary file 1 — Supplementary Material [file OPEN-14-e202500140-s001.pdf]

Supporting Information

High-Performance Oxidation and Nanomolar Detection of Phenylhydrazine Using a 6-Hydroxyflavone-Based Molecular Electrocatalyst Functionalized MWCNT in Batch Injection Analysis

V.Lavanya[a,b], K.Santhakumar[b]* and Annamalai Senthil Kumar[a,b]*

[a]*Nano and Bioelectrochemistry Research Laboratory, Carbon dioxide and Green Technology Research Centre, Vellore Institute of Technology University, Vellore – 632 014, Tamil Nadu,*

[b]*Department of Chemistry, School of Advanced Sciences, Vellore Institute of Technology University, Vellore – 632 014, Tamil Nadu, India*

Corresponding Author's E-mails: askumarchem@yahoo.com & askumar@vit.ac.in (A.S. Kumar) and phone number; +91-416-2202754

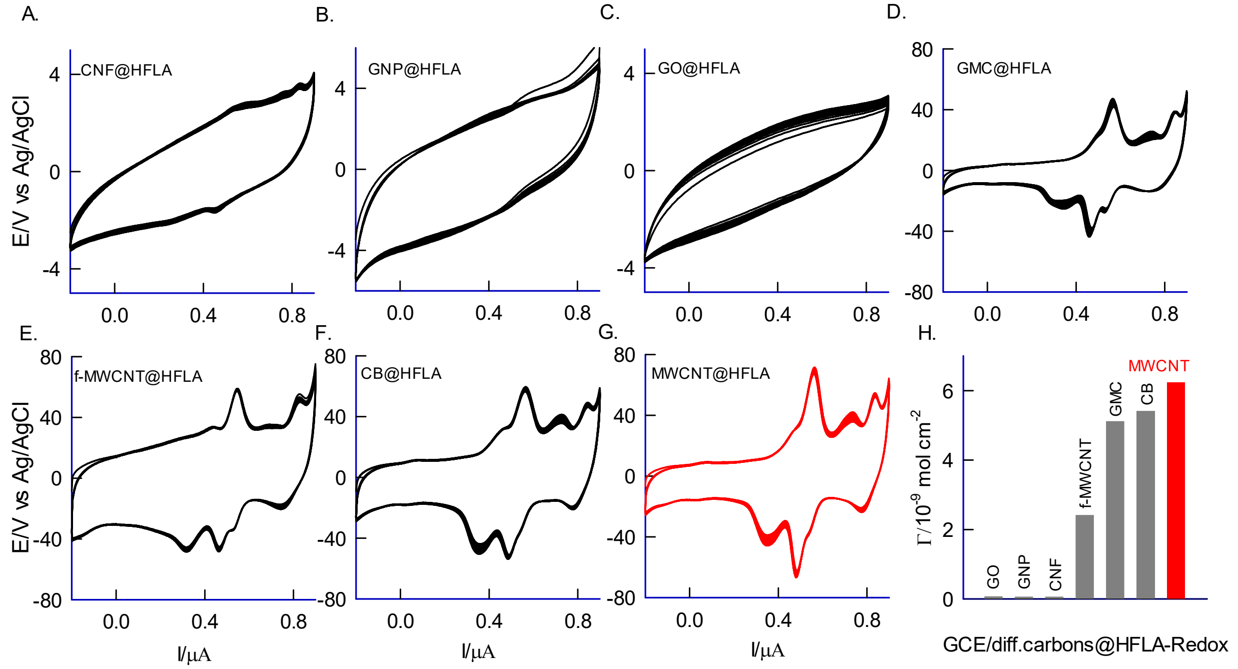

Figure S1. Effect of different carbon surfaces on the HFLA. Comparative CV responses of CNF (A), GNP (B), GO (C), GMC (D), f-MWCNT (E), CB (F) and MWCNT (G) for the formation of surface confined HFLA-Redox in pH 2 KCl-HCl at v=50 mV s^{-1}. (H) A plot of surface excess vs different carbon-based CME. **Note:** GO = Graphene Oxide; CNF = Carbon Nano Fiber; CB = Carbon Black; GNP = Graphene Nano Powder; GMC = Graphene Mesoporous Carbon; f-MWCNT = Functionalized Multi-Walled Carbon Nano Tube; MWCNT = Multi-Walled Carbon Nano Tube.

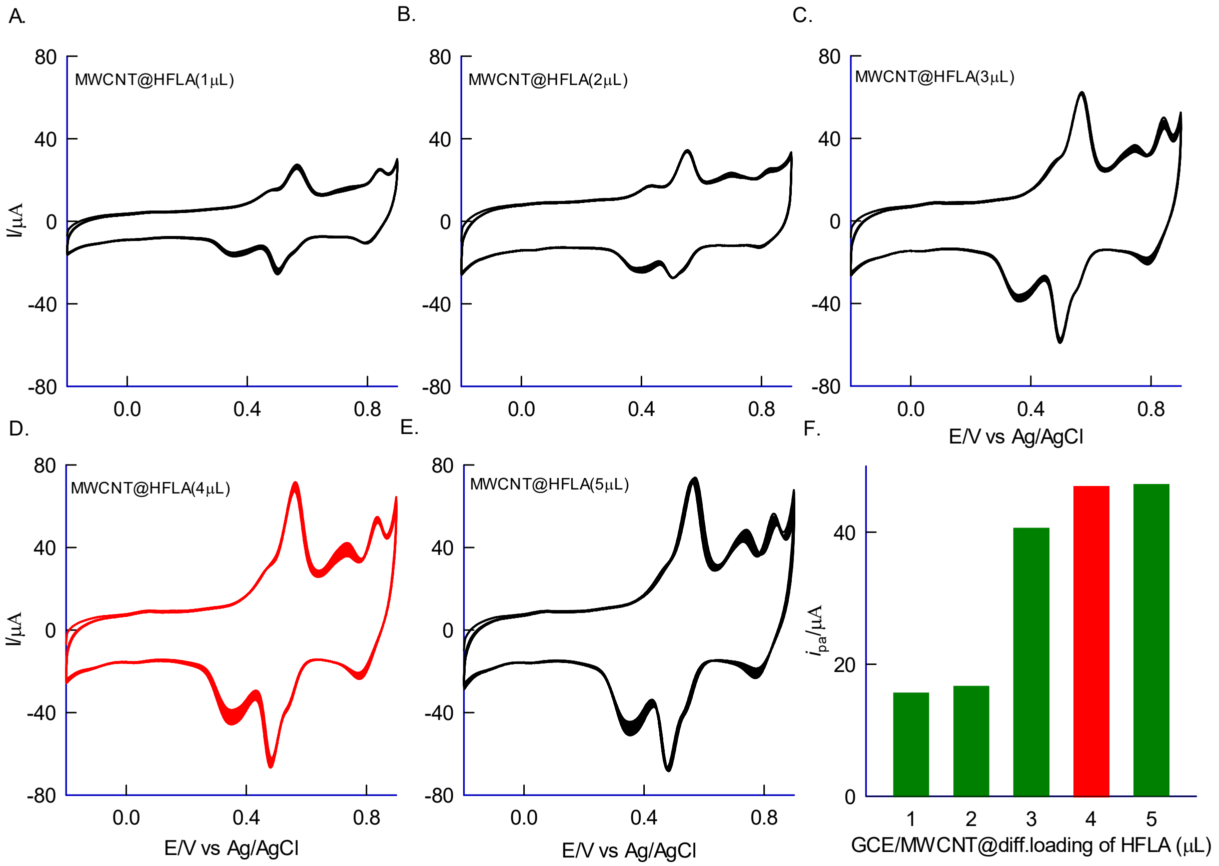

Figure S2. Effect of HFLA loading. CV responses of GCE/MWCNT at different loading of HFLA (A-E) for the formation of chemically modified electrode and (F) comparison of i_{pa} vs Diff. Loading of HFLA on GCE/MWCNT.

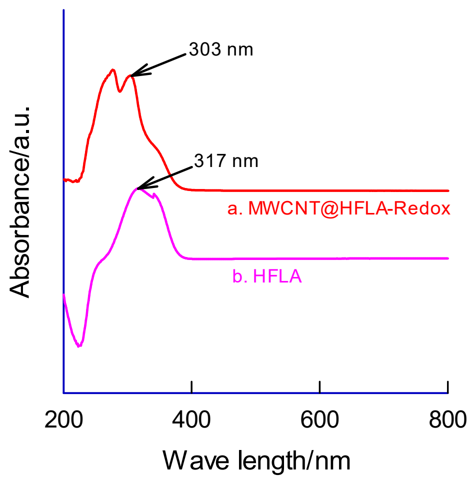

Figure S3. UV responses of HFLA-Redox (Ethanol extracted) (a), and HFLA (b)

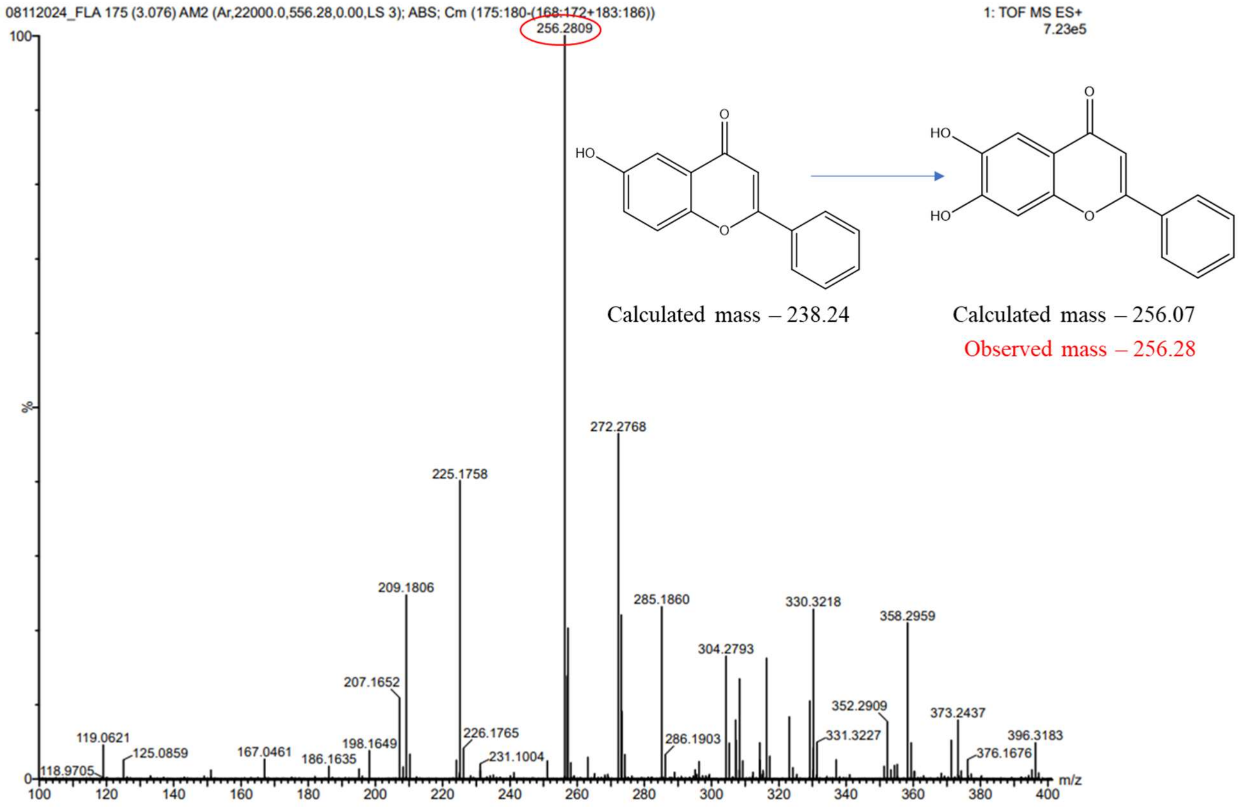

Figure S4. HR-MS analysis of methanolic solution of HFLA-Redox (isolated species). Inset is the plausible molecular structure and mass of the dione species.
